# A Rare Central Venous Catheter Malposition in a 10-Year-Old Girl

**DOI:** 10.1155/2018/2658640

**Published:** 2018-01-23

**Authors:** Ali Movafegh, Alireza Saliminia, Reza Atef-Yekta, Omid Azimaraghi

**Affiliations:** Department of Anesthesiology and Critical Care, Dr. Ali Shariati Hospital, Tehran University of Medical Sciences, Tehran, Iran

## Abstract

Central venous catheters (CVCs) are placed in operating rooms worldwide via different approaches. Like any other medical procedure, CVC placement can cause a variety of complications. We report the case of an unexpected malposition of a catheter in the right internal jugular vein, where it looped back on itself during placement and went upward into the right internal jugular vein. CVC line placement should always be viewed as a procedure that could become complicated, even in the hands of the most experienced operators.

## 1. Introduction

Central venous catheterization plays an important role in modern medical practice.

It is estimated that approximately 8% of hospitalized patients require central venous access during the course of their hospital stay, and it has been estimated that more than five million CVCs are inserted in patients in the United States each year [1, 2]. Indications for CVC placement are diverse. Some of the more common indications include invasive hemodynamic monitoring, parenteral nutrition support, dialysis, chemotherapy, fluid resuscitation, drug administration, and renal replacement therapy.

Although CV catheterization is a simple and relatively safe procedure, many complications have been reported during or after the procedure. Malposition is one of the complications observed.

In this report, we describe a rare case of malposition in a 10-year-old girl.

## 2. Case Presentation

The patient was a 10-year-old girl, recently diagnosed with leukemia and hospitalized for treatment. She required central venous (CV) line placement for chemotherapy. Other than her diagnosed leukemia, she had no other significant medical history.

After the procedure was explained thoroughly to the patient and her parents, a consent form was completed by her parents, and she was transferred to the CV line room. The patient was very alert and cooperative. Based on the local protocols, all patients requiring CV line placement are transferred to a room dedicated for CV line placement (the intravenous access room), which is located in the operating room. Standard monitoring including an electrocardiogram (ECG), noninvasive blood pressure, and pulse-oximetry were initiated. A 20-gauge cannula was inserted into the vein on the dorsum of the patient's left hand.

The right internal jugular vein was selected for CV cannulation.

Propofol was used for sedation, and after adequate sedation, a single-lumen 14-gauge catheter was inserted in the right internal jugular vein using ultrasound sonography under sterile conditions by an experienced anesthesiologist. No problem was encountered during the procedure, and after blood was aspirated, the catheter was fixed at 13 cm. Normal saline infusion was initiated through the CV. Central venous waveforms were not used for catheter position confirmation.

A chest radiograph was immediately arranged to confirm the catheter position.

**Figure 1:**
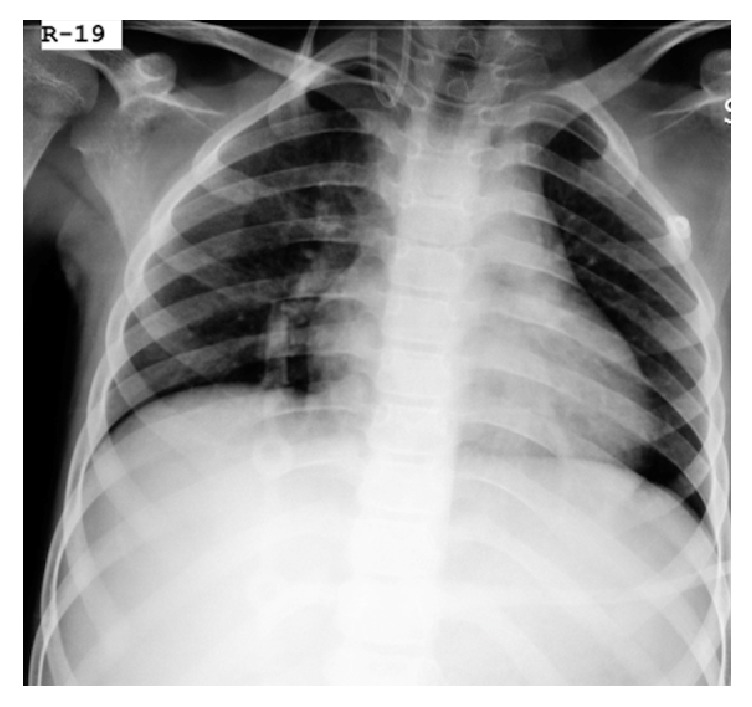
The catheter can be seen looping back and going upward at the junction of the right internal jugular vein and the right subclavian vein.

**Figure 2:**
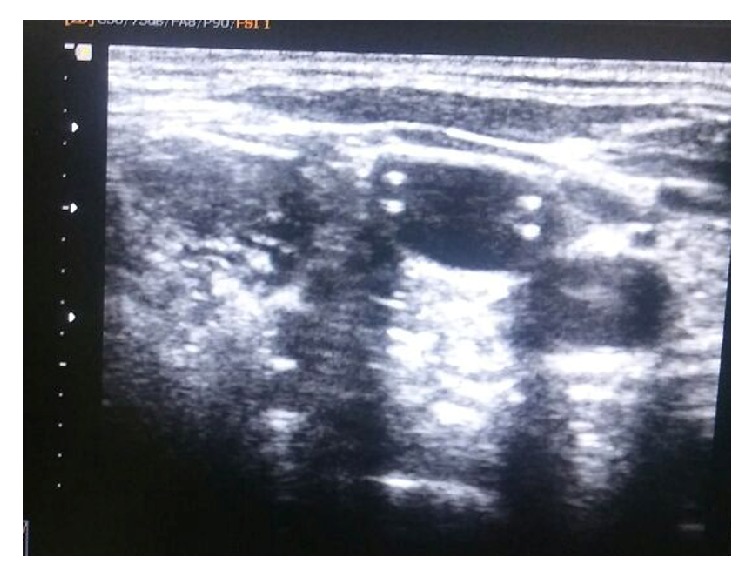
The right internal jugular vein is clearly evident on ultrasonography. Two lumens of the catheter can also be seen.

On chest radiograph, the catheter could be seen looping back and going upward at the junction of the right internal jugular vein and the right subclavian vein (Figure 1). The team decided to use ultrasonography to verify that the catheter was in the jugular vein and had not punctured the dorsal wall of the vein. During scanning of the right internal jugular vein, only a single lumen of the catheter could be seen until we scanned the bottom third of the jugular vein (Figure 2).

We decided to pull back the catheter under the guidance of ultrasonography until only one lumen could be visualized and then pass a guidewire over the catheter to reposition the catheter. While doing so and after only one lumen was visualized following extraction of the central venous line under the guidance of ultrasonography, passage of the guidewire was attempted, but resistance was encountered. Therefore, we decided to completely remove the catheter and reinsert another catheter.

**Figure 3:**
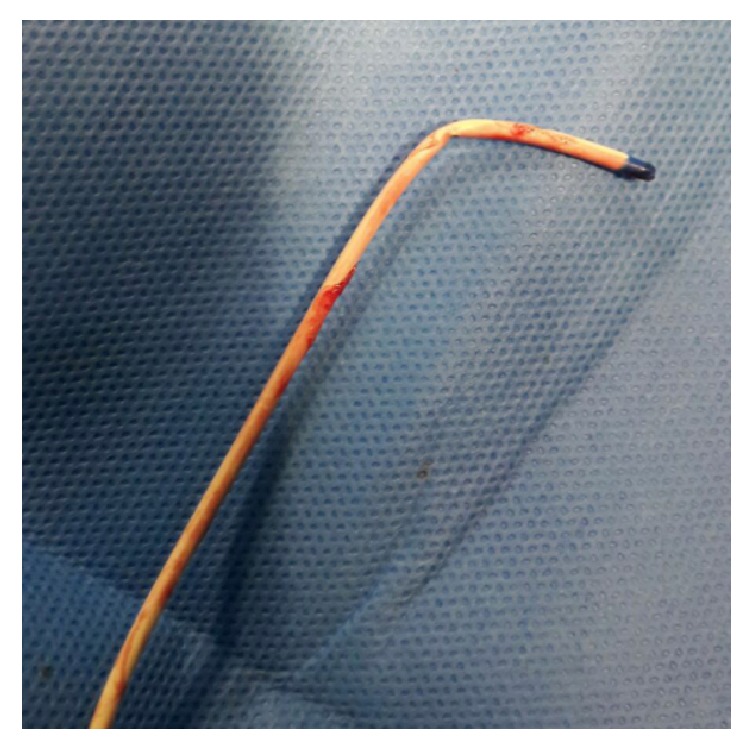
The bended catheter.

When the catheter was removed, it was observed that the catheter had bent at the distal end (Figure 3).

Subsequently, we placed another CVC through the internal jugular vein without any complications. The position of the CVC was confirmed by radiograph.

## 3. Discussion

CV catheterization is being performed every day in medical centers around the world. The internal jugular vein is one of the most common sites that anesthesiologists use, and this site is chosen because CVC can be securely inserted in this location.

It is difficult to estimate the rate of early and late complications that occur during insertion of CV lines. Many of the complications may go unseen, and many are unreported. Some complications may be life threatening or may cause morbidity, and some may not be recognized as a complication at all [3]. The incidence and occurrence of complications depend on various factors, such as the experience of the operator, the site of insertion, and the placement technique [4].

At our center, Shariati Hospital, we insert over 2000 CV lines every year. The Hematology-Oncology Research Center and Stem Cell Transplantation (HORCSCT) Center, which is affiliated to Tehran University of Medical Sciences (TUMS), is based in Shariati Hospital, and over two-thirds of our patients are individuals who have hematological cancer and require CV line placement for chemotherapy.

The low price of CV lines compared to other options for CV access has favored their use at our center. However, using CV lines for chemotherapy has its own hazards, especially in the pediatric population.

We use single-lumen 14-gauge CV lines for all our patients (aged 2 months and above), and the main route of insertion and technique of insertion for adults at our center is the subclavian vein via the landmark technique. Regarding the route and technique of insertion, we have found that patients are much more satisfied and can handle their daily tasks much easier when the catheter is fixed at a level below the clavicle.

In the pediatric patients, we usually use an ultrasound-guided approach to the right internal jugular vein, as we are inserting a very large catheter and the risk of complications is much higher via the subclavian vein approach.

In the case described here, the procedure went smoothly with no resistance, and the blood was successfully aspirated at the end of the first attempt at placement, which led us to refrain from scanning the catheter placement with ultrasound. This example shows that although insufficient blood flow from the catheter during aspiration is a possible warning sign of misplacement of the CV line, adequate blood aspiration cannot be totally relied on as a sign of successful placement.

Ultrasonography has aided in the placement of CVCs in many ways, especially in pediatric patients. However, as can be seen in this case, relying on ultrasound only for finding the vein and guiding the needle is not enough and did not prevent the malposition of the catheter. Based on this report, it is advised to scan along the vein to localize the catheter, even after a seemingly uneventful catheter placement. This is especially important when placing catheters via the subclavian vein, which is a site where a catheter risks going upward into the internal jugular vein.

Chest radiographs have always helped us determine early and late complications. The immediate chest radiograph that was taken in this patient proved to be vital in showing the complication.

Although we have had different types of malposition of catheters, this is the first time that we encounter a catheter looping back on itself. A probable mechanism could have been the angle of the internal jugular vein and subclavian vein, facilitating the malposition and pushing the j-shaped tip of the guidewire upward and back on itself. The right-sided bevel of the needle at the time of internal jugular vein puncture and guidewire insertion may also have contributed to the looping back of the guidewire and, consequently, the catheter.

Another issue that needs to be highlighted is the need for a specific facility for intravenous access procedures. Due to the large number of CVCs placed at our center, a few years ago we decided to dedicate a room with trained personnel for CV placement. Having a dedicated room for this purpose has not only decreased the time spent during each procedure but also decreased the rate of complications and increased patient satisfaction.

A variety of rare complications such as perforation of the left brachiocephalic vein and massive hemothorax, chylothorax, internal mammary artery malposition of catheter, and inadvertent placement of a CVC in the left pericardiophrenic vein have been reported previously [5–8].

It should be noted that a bending catheter has the risk of occlusion or perforation of the vein which fortunately did not occur in the described report above.

Many practical techniques such as using surface landmarks for estimating the length of catheter insertion, ultrasound-guided localization of the vein and guidance of the needle, echocardiography, electrocardiographic guided catheter tip placement using NaHCO3-filled catheters, and immediate postprocedure X-rays have been proposed for aiding a safe placement of CVC [9, 10].

We believe that to decrease the rate of complications associated with CVC placement, a multimodal approach is required. An appropriate setting with trained personnel, in combination with ultrasound guidance during and after the procedure, is helpful but not enough. Making sure the operator is focused throughout the procedure with attention to any atypical events such as resistance during any of the stages of catheter placement may help to decrease complications.

CV line placement should always be looked on as a procedure that could become complicated, even in the hands of the most experienced operators.

Therefore, it should be remembered that follow-up and checking of the correct function and placement of the CVC are as important as the procedure itself.
